# Endo-Aortic vs. Trans-Thoracic Clamping in Right Mini-Thoracotomy Mitral Valve Surgery: Outcome on Myocardial Protection

**DOI:** 10.3389/fcvm.2021.719687

**Published:** 2021-09-09

**Authors:** Cristina Barbero, Mauro Rinaldi, Marco Pocar, Erik Cura Stura, Claudia Calia, Viviana Sebastiano, Giovanni Marchetto, Claudia Filippini, Massimo Boffini, Davide Ricci

**Affiliations:** ^1^Department of Cardiovascular Surgery, Città della Salute e della Scienza, University of Turin, Turin, Italy; ^2^Department of Clinical Sciences and Community Health, University of Milan, Milan, Italy; ^3^Department of Anesthesia and Critical Care, University of Turin, Turin, Italy; ^4^Cardiac Surgery Unit, Scientific Institute for Research, Hospitalization and Healthcare Policlinic Hospital San Martino, Genova, Italy; ^5^Department of Integrated Surgical and Diagnostic Sciences, University of Genova, Genova, Italy

**Keywords:** myocardial protection, minimally invasive cardiac surgery, mitral valve, mitral valve repair, cardioplegia, aortic clamping, cardiopulmary bypass

## Abstract

**Background:** Perfusion strategies and aortic clamping techniques for right mini-thoracotomy mitral valve (MV) surgery have evolved over time and remarkable short- and long-term results have been reported. However, some concerns have raised about the adequacy of myocardial protection during the minimally invasive approach, particularly with the endo-aortic clamp (EAC). Aim of this study was to compare the efficacy, in terms of myocardial preservation, of the EAC with the trans-thoracic aortic clamp (TTC) in patients undergoing right mini-thoracotomy MV surgery.

**Methods:** A single center, prospective observational study was performed on patients undergoing right mini-thoracotomy MV surgery with retrograde arterial perfusion and EAC or TTC. A propensity matched analysis was performed to compare the two groups. Primary outcome was the comparison between cardiac troponin T levels measured at different time-points after surgery.

**Results:** Eighty EAC patients were compared with 37 TTC patients. No cases of myocardial infarction or low cardiac-output syndrome were overall reported. No differences were recorded in terms of stroke, peri-operative mortality, and in the release of myocardial markers, lactates levels and need for inotropic support at different time-points after surgery. CK-MB peak levels were significantly lower in the EAC group.

**Conclusion:** Despite concerns arising about the EAC, this prospective study shows equivalence in terms of myocardial preservation of the EAC compared with the TTC in patients undergoing right mini-thoracotomy MV surgery.

## Introduction

Perfusion strategies and aortic clamping techniques for right mini-thoracotomy mitral valve (MV) surgery have evolved over time and remarkable short- and long-term results have been reported all over the world (1–5). However, across the years, some concerns have emerged on the adequacy of myocardial protection offered by the minimally invasive approach and on the role of aortic clamping strategies in this context.

The standard right mini-thoracotomy set-up is based on retrograde arterial perfusion (RAP) through the femoral artery and endo-aortic clamp (EAC) or trans-thoracic aortic clamp (TTC). In the former, aortic occlusion is obtained with a balloon catheter inserted through the femoral cannula, and cardioplegia is delivered through the tip of the catheter; in the latter, aortic occlusion is obtained with a trans-thoracic direct clamping and a separate line into the ascending aorta is used for cardioplegia delivery. Efficacy of the EAC setting on myocardial preservation is definitely more debated due to several factors, including the higher risk of instability and possible migration or tearing of the balloon, and the more challenging learning curve for the surgeon (6).

Aim of this prospective observational study was to compare the effectiveness, in terms of myocardial protection, of the EAC vs. the TTC setting in patients undergoing right mini-thoracotomy MV surgery.

## Patients and Methods

### Study Design

A single-institution prospective observational study was performed at the University of Turin, Cardio-thoracic Department, between June 2014 and June 2018. The study protocol was reviewed and approved by the Institutional Ethics Committee (protocol number 0095187). Inclusion criteria were diagnosis of MV regurgitation, planned surgery through the right mini-thoracotomy and RAP. Exclusion criteria were non-elective surgery, cardiac ejection fraction lower than 40%, more than mild aortic regurgitation, previous coronary artery bypass grafts, coronary artery disease, severe peripheral vascular disease, and concomitant procedures for atrial fibrillation ablation. Patients admitted at our Department during the study period underwent a full preoperative work-up based upon clinical history and analysis of the vascular anatomy with the aim to be allocated to the most appropriate setting: RAP with EAC, RAP with TTC, or antegrade arterial perfusion with TTC (7–9). All comers were screened for the clinical trial inclusion criteria.

Primary outcome was the comparison between cardiac troponin T (cTn-T) levels measured at different time-points after surgery; secondary outcomes were peri-operative myocardial infarction, creatinine kinase-myocardial band (CK-MB) and lactates levels at different time-points after surgery, the occurrence of low cardiac output syndrome, and the need for post-operative inotropic support.

### Definitions

Peri-operative myocardial infarction was recorded in the case of cTn-T value >10 times the 99th percentile of the upper reference limit during the first 48 h with electrocardiographic (ECG) abnormalities and/or angiographic or imaging evidence of new myocardial ischemia/new loss of myocardial viability (10).

Low cardiac-output syndrome was defined as cardiac index lower than 2 L/min/m^2^ and systolic blood pressure lower than 90 mmHg, together with signs of tissue hypoperfusion (cold periphery, clammy skin, mental confusion, reduced urine output, elevated lactate levels) in the absence of hypovolemia (11).

Inotropic support was administered with the primary goal of maintaining a mean arterial pressure above 60 mmHg and cardiac index more than 2 L/min/m^2^. Vasoactive-inotropic score (VIS) indicates the amount of cardiovascular support by various inotropes or vasopressors in the first 24 h after the surgical procedure. The VIS was calculated as: dopamine dose (mg/kg/min) + dobutamine dose (mg/kg/min) + 100 × epinephrine dose (mg/kg/min) + 10 × milrinone dose (mg/kg/min) + 10.000 × vasopressin dose (unit/kg/min) + 100 × norepinephrine dose (mg/kg/min) (12).

Peri-operative mortality was defined as all deaths occurring during hospitalization or within 30 days of the procedure. Post-operative stroke was defined as a new, permanent neurological disability or deficit.

### Surgical Procedure and Myocardial Protection

Right mini-thoracotomy approach with perfusion strategies and aortic clamping techniques used in our Department has been previously described (8, 9). After full heparinization, peripheral cardiopulmonary bypass (CPB) was established with the patient cooled to 30°C. In the EAC setting, arterial cannulation was obtained with a 21F or a 23F cannula with sidearm (Edwards Lifesciences, Irvine, California); in the case of small arteries, a 21F cannula was used for inserting the EAC, while the contralateral artery was used for arterial perfusion by using a standard arterial cannula. In the TTC setting, a standard femoral cannula was used (18F or 20F, Edwards Lifesciences, Irvine, California). Venous return was routinely obtained with a double cannulation (jugular and femoral).

In the EAC setting, clamping and cardioplegia delivery were obtained through a balloon catheter (Intraclude®, Edwards Lifesciences, Irvine, CA) inserted through the sidearm of the arterial cannula; in the TTC setting, a Chitwood clamp, inserted through a first intercostal space port (Scanlan International, Inc., Minneapolis, MN, USA) was used and the cardioplegia was delivered through a 7F cardioplegia needle (CalMed Technologies, CA) placed into the ascending aorta. Antegrade myocardial protection was performed using St Thomas hospital solution 1 (Plegisol^TM^, Hospira Inc., Lake Forest, Illinois, USA) or Bretschneider solution (Custodiol®). At the end of the procedure air venting was obtained, in the EAC group, with the balloon inflated, through the cardioplegia line, while in the TTC group through the aortic line.

All patients receiving Bretschneider solution underwent minimal right atrium incision (<1 cm) in order to remove the crystalloid solution or underwent hemofiltration during cardiopulmonary bypass. No additional topical cooling, nor retrograde cardioplegia through the coronary sinus were used; aortic clamp was released above 33°C in all the patients.

After the surgical procedure, all patients were investigated for ECG abnormalities and for new hypokinetic or akinetic area by echocardiography.

### Biochemical Analysis

Blood samples for biochemical examination were collected immediately after the surgical procedure and at 6, 12, and 24 h post-operatively. Upper reference limit for CK-MB and for cTn-T were 5 ng/mL and 50 ng/L, respectively.

### Statistical Analysis

Continuous data are presented as mean and standard deviation (SD) or median and interquartile range (IQR) depending on data distribution, while categorical data are presented as rate and proportion. In univariate analysis continuous variables were compared using unpaired *t*-test or Wilcoxon-Mann-Whitney according to distribution type. Categorical variables were compared with the use of the Chi-square test or Fisher exact test, when appropriate.

To reduce possible differences between patients receiving EAC or TTC and obtain unbiased estimation of the treatment effect, a matched analysis using propensity score was performed. The propensity score of receiving EAC or TTC was estimated using a multivariable logistic regression analysis with the type of clamping as dependent variable. The priori selected variables were: age, gender, body mass index, pre-operative creatinine, peripheral vasculopathy, EuroSCORE II, previous cardiac surgery, ejection fraction, type of MV disease, length of aortic clamping, type of cardioplegia, and mean pressure during CPB.

The genetic matching method without replacement was used to match patients undergoing surgery with EAC or TTC (13–15). Standard differences were used to test balance and to compare the difference in the covariate between the pre- and post-matched samples, since the calculation is not dependent on sample size. A standardized difference is the difference in means between groups, divided by the (pooled) standard deviation: values <0.1 indicate adequate balance (16).

A mixed-linear regression model for repeated measures was used to examine the temporal effect across groups (EAC and TTC) during the 24-h observation period after surgery. Statistical tests were two-sided, and *p*-values of 0.05 or less were considered statistically significant and were conducted using the following software packages: Stata (Stata-Corp, College Station, Texas), R (R Foundation for Statistical Computing, http://www.r-project.org/) and SAS (SAS Institute, Cary, NC).

A receiver operating characteristic curve (ROC analysis) with peak cTn-T and CK-MB levels was performed. The results were expressed by providing the value of the AUC and relative interval at a 95% confidence level.

## Results

Five-hundred and sixty-six patients underwent right mini-thoracotomy MV surgery at our Department during the study period. One-hundred and eighty-nine patients met the study inclusion criteria. Of these, 81 underwent surgery with the EAC setting and 106 with the TTC setting; two patients did not undergo myocardial protection analysis due to conversion to median sternotomy before CPB onset. Groups were not comparable with respect to age, pre-operative creatinine level, peripheral vascular disease, EuroSCORE II, previous cardiac surgery, and type of MV regurgitation (Table 1). After matching, 80 EAC patients were compared with 37 TTC patients (Table 1). Standardized difference showed an adequate balance between groups regarding patients' baseline characteristics and intraoperative data apart from age, previous cardiac surgery, and mean pressure during CPB: in these cases, standardized difference between EAC and TTC widely reduced after matching, even if it still remains above 0.2.

**Table 1:** Patients' characteristics and operative data before and after matching.

	**Before matching**			**After matching**		
	**EAC** **(*n* = 81)**	**TTC** **(*n* = 106)**	**Standardized difference**	**EAC** **(*n* = 80)**	**TTC** **(*n* = 37)**	**Standardized difference**
Age years, mean (SD)	55.5 (12.4)	68.6 (10.2)	1.15	55.2 (12.4)	61.8 (9.1)	0.46
Female, *n* (%)	29 (35.8)	46 (43.4)	0.16	28 (35.0)	11 (29.7)	−0.08
BMI, mean (SD)	24.5 (3.6)	24.4 (3.5)	−0.02	24.5 (3.6)	24.2 (3.0)	−0.01
Pre-op creatinine mg/dL, mean (SD)	0.9 (0.2)	1 (0.4)	0.41	0.9 (0.2)	0.9 (0.2)	0.09
Peripheral arterial disease, *n* (%)	6 (7.4)	38 (35.8)	0.74	5 (6.3)	3 (8.1)	0.15
Ejection fraction %, mean (SD)	62.2 (7.2)	61.8 (7.9)	−0.04	62.2 (7.2)	62.8 (8.1)	0.11
Previous cardiac surgery, *n (%)*	9 (11.1)	1 (0.9)	−0.44	8 (10)	1 (2.7)	−0.26
Log EuroSCORE II, mean (SD)	4.3 (8.04)	5.7 (4.4)	0.20	4.0 (7.4)	3.0 (1.9)	−0.16
MV repair, *n* (%)	63 (77.7)	67 (63.2)	0.32	63 (78.8)	33 (89.2)	−0.20
Aortic clamping min, median (IQR)	99 (83–115)	89 (73–106)	−0.37	100 (83–115)	94 (83–113)	−0.19
Custodiol cardioplegia, *n* (%)	69 (85.2)	79 (74.5)	−0.27	69 (86.2)	33 (89.2)	−0.10
CPB pressure mmHg, mean (SD)	60.3 (5.7)	64.8 (6.2)	0.75	60.3 (5.7)	62.2 (5.5)	0.29

*EAC, endo-aortic clamp; TTC, trans-thoracic clamp; SD, standard deviation; BMI, body mass index; MV, mitral valve; CPB, cardio-pulmonary by-pass*.

No cases of EAC tearing or dislodgement were overall reported. No cases of myocardial infarction or low cardiac-output syndrome were observed. No ECG abnormalities or new hypokinetic or akinetic area were highlighted at the post-operative echocardiogram. No differences were recorded in terms of stroke, intensive care unit and hospital length of stay, and peri-operative mortality (Table 2). Two patients undergoing redo MV replacement in the TTC group died in 2nd and in 18th post-operative day, respectively. Cause of death were left ventricle rupture in a severe mitral annulus calcification in the first case, and multi-organ failure in the second case. Levels of myocardial markers in both the patients were below the median of the group.

**Table 2:** Outcomes in matched cohort.

	**EAC (*n* = 80)**	**TTC (*n* = 37)**	***p***
VF after aortic clamp release, *n* (%)	11 (13.8)	8 (21.6)	0.2830
Ventilation >72 h, *n* (%)	4 (5.0)	1 (2.7)	>0.9999
Stroke, n (%)	2 (2.5)	0 (0)	>0.9999
Myocardial infarction, *n* (%)	0 (0)	0 (0)	–
cTn-T peak level, mean (SD)	1016.8 (654.7)	1187 (744.5)	0.1071
CK-MB peak level, mean (SD)	1394.4 (668.4)	1775.6 (930)	0.030
Low cardiac output syndrome, *n* (%)	0 (0)	0 (0)	–
Dialysis, n (%)	1 (1.3)	1 (2.7)	0.5343
Re-exploration for bleeding, *n* (%)	0 (0)	1 (2.7)	0.3162
Post-op atrial fibrillation, *n* (%)	23 (28.9)	12 (32.4)	0.6858
ICU stay days, mean (SD)	1.6 (2.5)	1.3 (0.8)	0.3121
Hospital stay days, mean (SD)	7.4 (3.9)	6.8 (2.0)	0.3623
Peri-operative mortality, *n* (%)	0 (0)	2 (5.4)	0.0981

*EAC, endo-aortic clamp; TTC, trans-thoracic clamp; VF, ventricular fibrillation; cTn-T, cardiac troponin T; CK-MB, creatinine kinase-myocardial band; SD, standard deviation; ICU, intensive care unit*.

**Figure 1:**
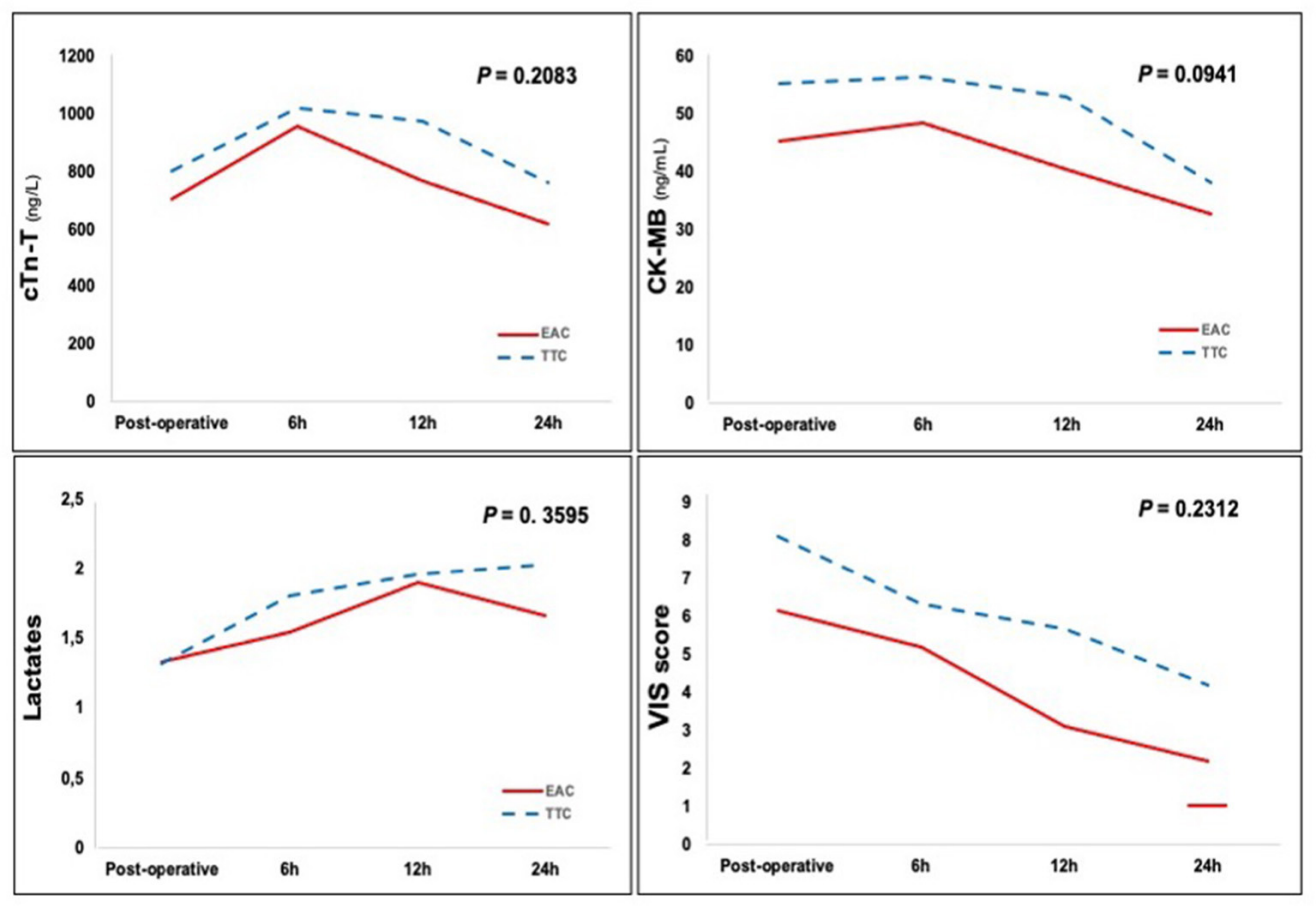
Post-operative cTn-T and CK-MB activities, lactates levels and VIS score. EAC, endo-aortic clamp; TTC, trans-thoracic clamp; cTn-T, cardiac Troponin T; CK-MB, creatinine kinase-myocardial band; VIS, vasoactive-inotropic score.

A significant lower CK-MB peak level was recorded in the EAC group (*p* = 0.030), while no differences were found in the release of myocardial markers, lactates levels and need for inotropic support at different time-points after surgery (Figure 1). The ROC analysis showed AUC = 0.56 (95% CI 0.48; 0.63) 95%, and AUC = 0.55 (95% CI 0.48; 0.63) 95% for post-operative peak level of CK-MB and cTn-T, respectively.

## Discussion

Adequate and safe myocardial protection represents the first key requirement in cardiac surgery, regardless of the type of surgical procedure and the approach. When looking at right mini-thoracotomy MV surgery, concerns still remain about the role of aortic clamping strategies, particularly the EAC, on the risk of sub-optimal myocardial protection. The rationale is that it requires consolidated team experience to get the right position and pressure of the balloon in the ascending aorta and to maintain those conditions through all the procedure; migration of the balloon toward the left ventricle or toward the aortic arch, or accidental balloon puncture or tearing could be responsible for inadequate myocardial protection. For these reasons, the TTC is preferred to the EAC in many centers. However, it requires some kind of aortic manipulation, such as dissection around the ascending aorta to allow the external clamp positioning and a separate line for cardioplegia delivery; these maneuvers, compared to the “aortic no touch technique” of the EAC, increase the risk of local damage to the aorta, and some degrees of calcium embolization (6, 17).

A propensity matched analysis comparing the EAC with the TTC in terms of myocardial protection was performed at our Department using established markers of myocardial injury, i.e., cTn-T and CK-MB. Results of the present study do not support concerns existing in the literature regarding the EAC setting: equivalence at each time point after surgery was recorded for cTn-T levels, lactates levels and VIS score; moreover, a significant lower CK-MB post-operative peak level was recorded in the EAC group (*p* = 0.030). The area under the curve for cTn-T has demonstrated excellent linear relationship with the mass of myocardial injury; the area under the curve for CK-MB is also good, although clearly inferior to cTn-T (18). Therefore our results can be read as equivalence between the two aortic clamping techniques.

During the study period and during our every-day clinical practice the choice of one setting in respect to the others is orientated on the patient's aorto/iliac/femoral anatomy and clinical history, regardless of the surgeon's preference. Such as, in case of previous cardiac surgery the EAC setting is used mostly; in case of dilated ascending aorta (diameter >40 mm) the TTC setting was predominantly used; in case of aorto-iliac-femoral arteries disease, an antegrade arterial perfusion with TTC was preferred (8, 19, 20). In cases of patients without peripheral vessel disease or other main comorbidities the setting of choice in our practice is RAP with EAC because it does not require to open extensively the pericardium in order to get a full exposure of the ascending aorta, it does not require to place a cardioplegia/vent needle into the ascending aorta, and it does not require getting a further port in the first intercostals space to introduce and place the aortic clamp (8, 19).

Results of the present study are in contrast with reports of other centers showing superiority of the TTC. Mazine et al., in a retrospective analysis on 243 patients undergoing right mini-thoracotomy MV surgery and EAC or TTC concluded that the TTC setting results in better myocardial protection (6). However, several bias may have influenced this result: longer operative time and several cases of endoclamp dislodgement were reported in the EAC group; cases from both groups were not evenly distributed in time (the balloons were mostly used at the beginning of the experience and thus at the beginning of the learning curve); furthermore only CK-MB plasma levels were taken into consideration for myocardial injury analysis.

More recent experiences show equivalence or even better results with the EAC setting. Bentala et al., in a retrospective analysis on 221 patients undergoing right mini-thoracotomy MV surgery and EAC or TTC showed comparable results in terms of post-operative myocardial infarction and lower maximum post-operative cTn-T levels with the EAC setting (21). A multicentric European study on 500 right mini-thoracotomy MV surgery patients reported a significant lower rate of post-operative myocardial infarction in the EAC group (1.2 vs. 4% in the TTC group, *p* = 0.05) (17). The improvement in myocardial protection in more recent series can be explained considering that these studies have been reported by surgeons with a well-established learning curve and with a consolidated program of patient selection based upon vascular anatomy and clinical history. Another potential influencing factor in the different rate of complications is the type of endo-balloon used: while most of the first reports on right mini-thoracotomy MV surgery with the EAC setting used the Endoclamp, in most recent series either the Endoclamp and the IntraClude devices were used with respect to the time of distribution of the products (Endoclamp at the beginning, Intraclude later). The Intraclude has different new features able to enhance manipulation and stabilization of the balloon in the ascending aorta during the surgical procedure.

The single-center non-randomized study design is clearly the main limitation. However, the reason for not performing a prospective randomized clinical trial was driven by our consolidated practice in minimally invasive surgery based on a tailored approach for each patient. Moreover, the propensity matched analysis reached a suboptimal balance between groups regarding age, previous cardiac surgery, and mean pressure during CPB. Standardized difference for these variables widely reduced after matching, even if it still remains above 0.2: this can be explain considering that in our every-day clinical practice the EAC is the preferred setting in redo cases and in young patients without atheromatous disease. Furthermore, with the EAC setting the mean pressure during CPB is more accurately verified and persistently kept low than 60–65 mmHg because it is one of the determinant factors in maintaining the right position of the balloon during the clamping time; this gives reasons for the different mean CPB pressure recorded between the two groups. It must be said that, from a clinical point of view, there are no differences between 60 and 62 mmHg.

## Conclusions

Despite concerns arising about the EAC, this prospective study shows equivalence in terms of myocardial protection of the EAC compared with the TTC in patients undergoing right mini-thoracotomy MV surgery.

## Data Availability Statement

The raw data supporting the conclusions of this article will be made available by the authors, without undue reservation.

## Ethics Statement

The studies involving human participants were reviewed and approved by Institutional Ethics Committee Città della Salute e della Scienza - Torino (protocol number 0095187). The patients/participants provided their written informed consent to participate in this study.

## Author Contributions

CB and MR: conceptualization. CB and DR: methodology. CF: software. MR, MB, MP, and GM: validation. CB and CF: formal analysis. ES, CC, and VS: investigation and data curation. CB: writing-original draft preparation and writing-review and editing. MR: supervision and project administration. All authors contributed to the article and approved the submitted version.

## Conflict of Interest

The authors declare that the research was conducted in the absence of any commercial or financial relationships that could be construed as a potential conflict of interest.

## Publisher's Note

All claims expressed in this article are solely those of the authors and do not necessarily represent those of their affiliated organizations, or those of the publisher, the editors and the reviewers. Any product that may be evaluated in this article, or claim that may be made by its manufacturer, is not guaranteed or endorsed by the publisher.
